# Long-term visual outcomes in children with regressed retinopathy of prematurity

**DOI:** 10.1038/s41598-023-31234-2

**Published:** 2023-03-11

**Authors:** Se Hie Park, Dae Joong Ma, Dong Gyu Choi

**Affiliations:** Department of Ophthalmology, Hallym University College of Medicine, Kangnam Sacred Heart Hospital, 665 Shiheongdae-Ro, Seoul, 07442 Korea

**Keywords:** Eye diseases, Risk factors

## Abstract

This retrospective study evaluated long-term visual outcomes in children with regressed retinopathy of prematurity (ROP) and correlations between visual acuity (VA) and clinical variables, including fundus findings. We reviewed the medical records of 57 consecutive patients diagnosed with ROP. We analyzed the correlations between best-corrected VA and anatomical fundus findings, such as macular dragging and retinal vascular tortuosity, after ROP regression. The correlations between VA and clinical variables such as gestational age (GA), birth weight (BW), and refractive errors (hyperopia and myopia in spherical equivalent [SE], astigmatism, and anisometropia) were also evaluated. Of 110 eyes, 33.6% had macular dragging; the presence of macular dragging and poor VA were significantly correlated (*p* = 0.002). Patients with larger macula-to-disc distance/disc diameter ratios had significantly poorer VA (*p* = 0.036). However, no significant correlation was observed between the VA and vascular tortuosity. Patients with smaller GA and BW had poorer visual outcomes (both, *p* = 0.007). The larger SE in absolute values, myopia, astigmatism, and anisometropia were significantly associated with poorer visual outcomes as well (all, *p* < 0.001). In children with regressed ROP, macular dragging, small GA and BW, large SE in absolute values, myopia, astigmatism, and anisometropia may be predictors of poor visual outcomes at early ages.

## Introduction

Retinopathy of prematurity (ROP) is a vision-threatening disease associated with abnormal retinal vascular development in premature infants. In the past decade, major advances have been made in the management of ROP. Conventional treatment options for ROP include cryotherapy and laser photocoagulation^1^. Although laser treatment remains the gold standard for threshold ROP, the efficacy and safety of anti-vascular endothelial growth factor (anti-VEGF) therapy have recently been discussed. The Bevacizumab Eliminates the Angiogenic Threat for Retinopathy of Prematurity (BEAT-ROP) trial showed the benefits of anti-VEGF agents in severe ROP^2^. Surgical treatment, including scleral buckling or vitrectomy, is needed in the advanced stages of ROP with retinal detachment.

However, eyes with sight-threatening stages of ROP can develop a range of ocular morbidities that are visually impairing even after the resolution of ROP. The vision impairment (VI) rate in ROP was high at 4.6 per 100 person-years in the very low birth weight (VLBW) population, and a greater association with VI was observed, especially in eyes that underwent scleral buckling or vitrectomy^3^. Moreover, refractive errors, such as myopia, hypermetropia, astigmatism, anisometropia, and strabismus, may cause ocular morbidities resulting in permanent VI, even in cases having stable retinal status with or without adequate management. Delayed detection and adequate management of these refractive errors can eventually lead to amblyopia. Anatomical sequelae, such as retinal vascular tortuosity or macular dragging after regression, may also occur. When the retina is dragged temporally, observed as the straightening and narrowing of the angle between the major vessels, it could also cause VI^4^.

To the best of our knowledge, there are few studies investigating the correlation between anatomical sequelae of ROP (especially macular dragging and vessel tortuosity) and visual outcome. We performed this study to predict the long-term prognosis of visual outcome in patients with regressed ROP after proper retinal management and factors related to poor visual outcomes.

## Materials and methods

### Study design and subjects

We retrospectively reviewed the medical records of 57 consecutive patients (110 eyes) who had been diagnosed and managed with ROP in the retina clinic at Kangnam Sacred Heart Hospital, Hallym University, Seoul, between 2010 and 2018, and consecutively followed up at the pediatric ophthalmology clinic until the ages at which visual acuity (VA) using Snellen chart and conventional fundus photography could be obtained. Some patients were transferred to our clinic from other hospitals after ROP treatment. Children with systemic diseases such as neurologic disease, Down’s syndrome, and cerebral palsy, which made VA unreliable, and those with structural abnormalities of the eyes unrelated to ROP were excluded from the initial review. We also excluded children who did not have medical records of Snellen VA or fundus photography. This study was performed in accordance with the tenets of the Declaration of Helsinki and approved by the Institutional Review Board (IRB) of Hallym University Medical Center (IRB no. 2022-05-015). The need to obtain informed consent was waived because of the retrospective study design and use of anonymized clinical data.

### ROP screening and management

In the neonatal intensive care unit, infants who weighed less than 1,500 g at birth or those who were less than 30 weeks of gestational age (GA) were screened for ROP^5–7^. Regarding the Screening Examination of Premature Infants for Retinopathy of Prematurity^5,6^, we classified patients by zone and stage of abnormal findings. Each of the three zones of the retina is centered on the optic disc. Zone I includes the posterior pole and is defined as a circle, centered on the disc, whose radius is twice the distance from the disc to the center of the macula. Zone II extends from the peripheral border of Zone I to a concentric circle tangential to the nasal ora serrata. Temporally, this boundary corresponds approximately to the anatomic equator. Zone III is the remaining temporal crescent of retina anterior to Zone II. Abnormal peripheral changes are divided into five stages, which may progress to retinal detachment; Stage 1: Demarcation line, Stage 2: Ridge, Stage 3: Ridge with extraretinal fibrovascular proliferation, Stage 4: Partial retinal detachment, Stage 5: Total retinal detachment. The presence of vascular changes with marked increasing dilation of the posterior vein and tortuosity of the arterioles, represents a plus disease which denotes a worse prognosis^8^. Certain patients were categorized as aggressive posterior ROP (AP-ROP), which is a severe form of ROP characterized by rapid progression to advanced stages in posterior ROP. Characteristic features of AP-ROP are abnormal retinal findings located in posterior retina Zone I or posterior Zone II, prominence of plus disease (in all four quadrants), and the ill-defined nature of the retinopathy such as neovascularization. Retinal examination was performed every 1–2 weeks until ROP regression and complete vascularization of the retina were observed. Patients who met the treatment indication were treated with laser photocoagulation and/or intravitreal anti-VEGF injection. According to the 2006^5^ and 2013^6^ American Academy of Pediatrics guideline, we performed laser therapy in the early period (before 2013) as a first-line treatment modality. Thereafter, since several studies, including the BEAT-ROP trial, suggested the benefits of intravitreal anti-VEGF monotherapy^2^, we commenced intravitreal bevacizumab injection combined with laser from 2013. Anti-VEGF injection is inexpensive, easier to perform, and less stressful to infants compared to laser therapy. Recently, according to the RAINBOW study^9^, of ranibizumab versus laser therapy for the treatment of very low birthweight infants with ROP, intravitreal ranibizumab injection monotherapy has been undertaken as first-line therapy. If ROP is not regressed after anti-VEGF treatment, laser therapy is then considered.

### Ophthalmologic examination

The retinal specialists referred the patients to the pediatric ophthalmology clinic if ROP regressed. If there were ongoing abnormal retinal findings at the age of one, they were recommended to visit both the retinal and the pediatric ophthalmology clinics. Cycloplegic refraction with 1% cyclopentolate, 1% tropicamide, and slit-lamp biomicroscopic and fundus examinations were performed. The deviation angle was measured using the alternate prism cover test at a distance and a near range (6 and 1/3 m) for all fields of gaze, using accommodative targets (with spectacle correction based on cycloplegic refraction, if necessary). The Krimsky method was used for uncooperative children.

VA was measured with Teller acuity cards initially. Once the children grew up to be cooperative, the optotype measurement was conducted with the Snellen acuity chart at a 5-m distance after optical correction based on cycloplegic refraction, if necessary. Patients with amblyopia underwent occlusion therapy with eyepatches.

### Analysis of fundus images

**Figure 1:**
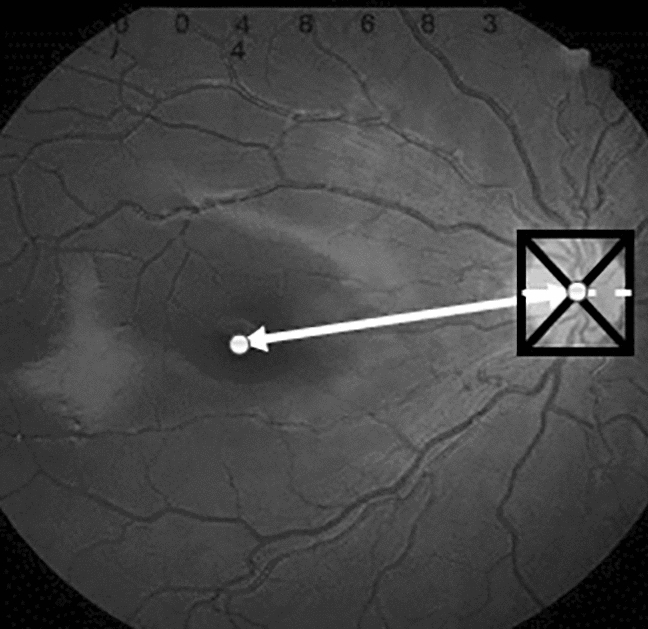
Macula-to-disc distance. A rectangle is positioned around the border of the optic disc. The center of the rectangle is determined by drawing lines from the corners of the rectangle. The short diameter of the neural tissue of the optic disk (DD, white dotted line) and the distance between the center of the disc and the center of the macula (DM, white arrow) are measured.

**Figure 2:**
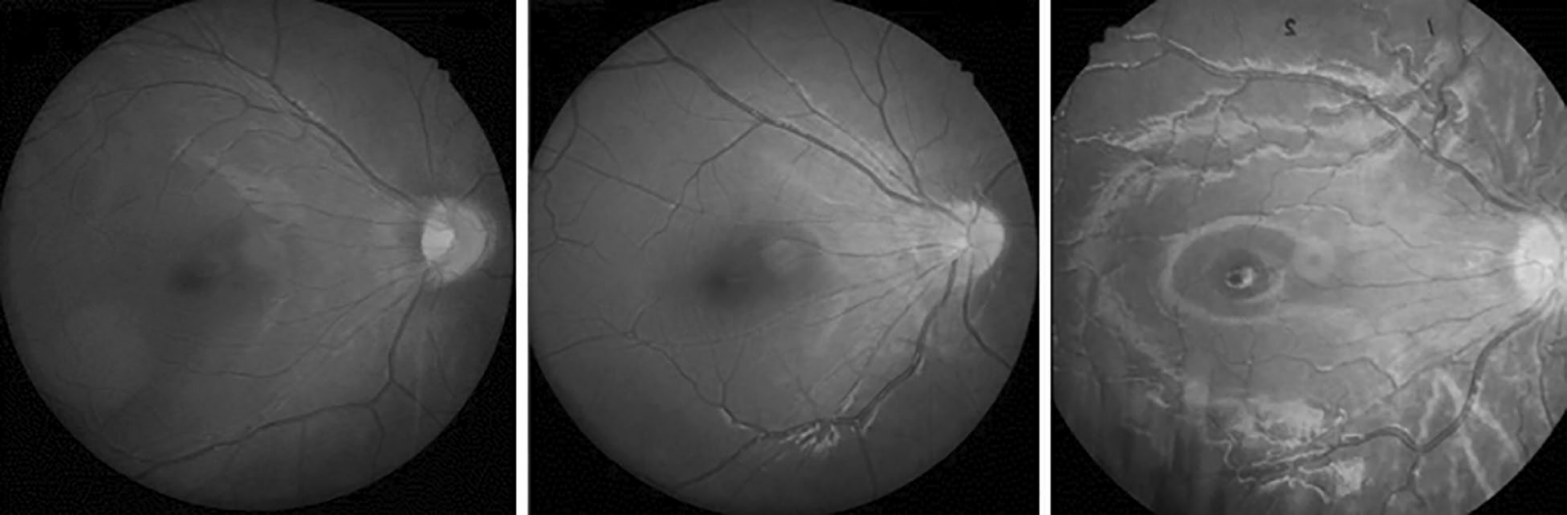
Macular dragging, straightening of the retinal vessels arising from the optic disc, and narrowing of the angle between major vessels are shown.

Fundus photographs were taken by one of four trained technicians using a digitalized fundus camera (KOWA Nonmyd 8S Fundus Camera, KOWA company, Japan) with pupil dilation during cycloplegic refraction, producing macular and optic disc images. The macula-to-disc distance was measured in pixels using an ImageJ scale-bar (ver.1.52; National Institute of Health, Bethesda, MD, USA), based on a method described by Hong et al.^3^ A rectangle was positioned around the border of the optic disc. The center of the rectangle was determined by drawing lines from its corners. The short diameter of the neural tissue of the optic disk (DD) and the distance between the center of the disc and center of the macula (DM) were measured (Fig. 1). The DM/DD ratio was used to assess the relative macula-to-disc distance. A DM/DD ratio > 3 or the presence of retinal folds with or without vessel straightening were considered “macular dragging” (Fig. 2).

**Figure 3:**
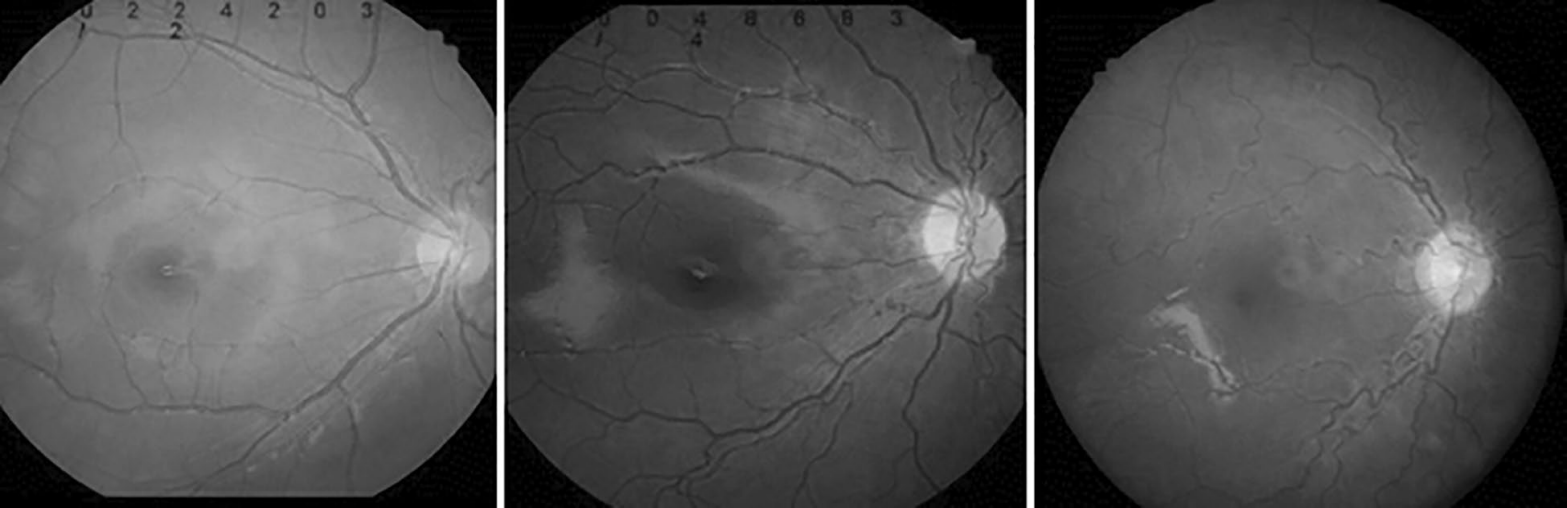
Vascular tortuosity: graded based on standard photographs selected by two ophthalmologists’ consensus (1, no tortuosity; 2, mild to moderate; 3, severe).

“Retinal vascular tortuosity” was graded based on standard photographs selected by consensus between one pediatric ophthalmologist (DGC) and one retinal specialist (DJM) (1, no tortuosity; 2, mild to moderate; 3, severe) (Fig. 3). We assessed the photographs masked to the corresponding clinical information. When the two observers’ conclusions were discordant, a third assessment was performed by an observer (SHP) masked to the results by the initial two observers. If the third assessment did not agree with any of the initial assessments, the three observers viewed the photographs together and tried to reach a consensus.

### Main outcome measures

The correlations between VA and anatomical fundus findings, such as macula-to-disc distance and tortuosity, were evaluated. Correlations between VA and several clinical variables, such as sex, age, GA, birth weight (BW), and refractive errors (hyperopia and myopia in spherical equivalent [SE], astigmatism, and anisometropia), were also evaluated. SE was defined as the diopter (D) value of the sphere plus that of astigmatism/2. Anisometropia was defined as the difference in SE of > 2D between the two eyes. For the analysis, the myopia group was defined as an SE of ≤ –1.0D and hyperopia as an SE of ≥  + 1.5D. If a patient had several VA records, the best VA results were used for the analyses.

### Statistical analysis

Both eyes of each subject were evaluated for analyses. However, if only one eye was diagnosed with ROP, only the affected eye was included. Spearman’s correlation tests were performed to investigate the association between ROP and potential risk factors. The interobserver reproducibility for vascular tortuosity grading was calculated according to the intraclass correlation coefficients (ICC) from a two-way mixed-effect model using SPSS Statistics. A coefficient of > 0.80 indicated an almost perfect agreement; > 0.60–0.80, substantial agreement; and > 0.4–0.6, moderate agreement. The measurement values for interobserver reproducibility were calculated by two observers (DGC and DJM). Statistical analyses were performed using IBM SPSS Statistics (version 22.0; SPSS Corp., Chicago, IL, USA). A confidence interval of 95% and corresponding *p* < 0.05 were considered statistically significant.

## Results

Of the 57 patients, ROP was diagnosed in both eyes in 53 patients and one eye in four patients; therefore, 110 eyes were included in this study. The ROP was regressed with or without treatment in all the subjects included in this study. Their demographic characteristics are listed in Table 1. The average GA was 27.55 ± 2.60 weeks (range, 23–34 weeks), and the mean BW was 1.06 ± 0.38 kg (range, 0.53–2.00 kg). The average age at which the VA measurements were used for the analyses was (5.2 ± 1.60) years (range, 2–9 years). Of the 110 eyes, 61.8% underwent laser coagulation only, 14.5% had additive intravitreal anti-VEGF injection, and 24% were not treated.

**Table 1:** Demographic characteristics of the enrolled subjects (N = 57, 110 eyes).

Variables	
Age (years)	5.2 ± 1.60 (2.40–9.00)
Sex	
Male/female [patients (%)]	29 (50.9)/28 (49.1)
Gestational age (weeks)	27.55 ± 2.60 (23–34)
Birth weight (kg)	1.06 ± 0.38 (0.53–2.00)
Strabismus [patients (%)]	15 (26.3)
Exotropia/esotropia	12 (21.0)/3 (5.3)
Refractive error [eyes (%)]	
Hyperopia/emmetropia/myopia	25 (22.8)/41 (37.2)/ 44 (40.0)
Astigmatism [eyes (%)]	79 (71.8)
Anisometropia [patients (%)]	15 (26.3)
ROP classification [eyes (%)]	
AP-ROP	20 (18.2)
Zone I	
Stage 3 + /3/2 + /2	8 (7.3)/2 (1.2)/2 (1.2)/2 (1.2)
Zone II	
Stage 3 + /3/2/1	27 (24.5)/2 (1.2)/4 (3.6)/13 (11.8)
Zone III	
Stage 2/1	2 (1.2)/1 (0.1)
Not specified	27 (24.5)
Treatment for ROP [eyes (%)]	
No treatment	26 (23.6)
Laser treatment only	64 (58.2)
Laser + intravitreal injection	20 (18.2)

Values are presented as numbers (%) or mean ± standard deviation (range). Myopia, spherical equivalent (SE) ≤  − 1.0 D; hyperopia, SE ≥  + 1.5D; astigmatism, cylindrical error (Cyl) ≤ -1.0 D without reference to the axis; anisometropia, SE difference between the two eyes > 2 D.

ROP = retinopathy of prematurity; AP-ROP = Aggressive Posterior ROP : located in posterior retina (Zone I or posterior Zone II), prominence of plus disease (in all four quadrants), and the ill-defined nature of the retinopathy.

Stage1 = Demarcation line; Stage2 = Ridge; Stage3 = Ridge with extraretinal fibrovascular proliferation.

Plus sign ( +) = vascular changes with marked increasing dilation of posterior vein and tortuosity of the arterioles.

A favorable visual outcome defined as 20/25 in the Snellen chart test (0.10 logMAR) or better was noted in 86 eyes (78.2%), while a poor visual outcome defined as worse than 20/160 (0.90 logMAR) was noted in only one eye (0.9%). The mean acuity was 0.09 ± 0.20 logMAR (range, 0–1.70) and mean SE was − 1.33 ± 3.19D (range, − 21.50 to + 5.50 D). Among 110 eyes, 44 were myopic (40.0%), 41 (37.2%) were emmetropic, and 25 (22.8%) were hyperopic. Fifteen patients (26.3%) had anisometropia, defined as a difference of SE ≥ 2.0 D between the two eyes.

Of the 15 patients (26.3%) who showed strabismus, exotropia was present in 12 with a mean deviation angle of 19.25 ± 8.6 prism diopter (PD) (range, 8–40) and esotropia in 3 with a mean deviation angle of 22.7 ± 11.7 PD (range, 8–30).

Of the 110 eyes, 37 (33.6%) had macular dragging. There was a statistically significant association between the presence of macular dragging and visual outcome (*p* = 0.002). Mean value of macula-to-disc distance divided by shortest disc diameter (DM/DD) was 3.05 ± 0.26 (range, 2.29–5.92). Patients with a larger DM/DD ratio tended to have poor visual outcomes with statistical significance (Spearman’s correlation coefficient: 0.200, *p* = 0.036).

Thirty eyes (27.3%) had normal retinal vascular contours without vascular tortuosity, 60 (54.5%) had mild to moderate tortuosity, and 20 (18.2%) had severe tortuosity. The ICC was 0.97, indicating almost perfect agreement between the observers. There was no significant correlation between best-corrected VA (BCVA) and vascular tortuosity.

Patients with lower GA and BW had poorer visual outcomes (*p* = 0.007 and 0.007, respectively). A larger SE in absolute values and anisometropia also led to poorer visual outcomes (*p* < 0.001). Correlation between BCVA and clinical variables are listed in Table 2.

**Table 2:** Spearman’s correlation analysis of best-corrected visual acuity (BCVA) and clinical variables.

Variables	Correlation coefficient	*P *value
Age	− 0.094	0.328
Sex	0.015	0.876
Gestational age	− 0.253	0.007
Birth weight	− 0.251	0.007
SE (absolute value)	0.434	< 0.001
Refractive error		
Myopia	− 0.358	0.000
Hyperopia	− 0.013	0.890
Astigmatism	− 0.334	0.000
Anisometropia	− 0.474	< 0.001
Retinal vascular tortuosity	− 0.113	0.241
Macular dragging	− 0.290	0.002
Macula-to-disc distance/disc diameter	0.200	0.036

Values are presented as numbers (%) or mean ± standard deviation (range). Myopia, spherical equivalent (SE) ≤  − 1.0 D; hyperopia, SE ≥  + 1.5D; astigmatism, cylindrical error (Cyl) ≤ -1.0 D without reference to the axis; anisometropia, SE difference between the two eyes > 2 D.

This study found that the patients in the treatment group (laser monotherapy or laser and intravitreal bevacizumab injection) had significantly poorer VA compared to the untreated group (*p* = 0.02). However, there was no significant correlation between treatment modalities (laser monotherapy versus laser and intravitreal bevacizumab injection) and VA.

## Discussion

According to a recent nationwide population-based study in South Korea, the incidence of ROP was one in 500 in the newborn population and one in three in the VLBW population^10^. The VI rate in ROP was high (4.6 per 100 person-years) in the VLBW population. The importance of ROP evaluation and treatment is growing as preterm birth survival, especially VLBW survival, is increasing over time^10^.

The development of ROP treatment, especially the shift in the treatment paradigm from primary laser to primary anti-VEGF agents, has been known to decrease adverse structural outcomes, high myopia, cataracts, and glaucoma^11^. However, residual sequelae after ROP regression can lead to long-term VI^12^. After the regression of acute-phase ROP, a wide range of structural changes in the retina and retinal vessels may follow^6^. Mild sequelae such as persistence of retinal vascular tortuosity, peripheral pigmentations, and vascular anomalies are known to have less impact on visual function, while changes such as macular dragging, distortion, or detachment can induce severe VI^4^.

Our study demonstrated a correlation between visual outcome and retinal structural changes. Thirty-seven eyes (33.6%) had macular dragging, which was significantly associated with poor visual outcomes. Moreover, there was a correlation between VA and severity of macular dragging, which was assessed using the DM/DD ratio.

These results may be clinically important to pediatric ophthalmologists. Since we can easily obtain conventional fundus photographs at an early age or RetCam images even in infants, structural findings can aid in predicting approximate visual prognosis in young children after ROP regression; however, VA measurement and retinal imaging tests such as wide fundus photography and optical coherence tomography are difficult to obtain and unreliable. Another retinal finding in ROP is the persistent retinal vascular tortuosity. In this study, 72.7% of the eyes had vascular tortuosity, which did not significantly correlate with the visual outcome.

Even when the retina was evaluated to be normal, other clinical variables, such as strabismus, amblyopia, and perinatal neurological events, are known to cause poor visual outcomes. Yang et al. reported that 1.7% of the treated eyes had abnormal retinal structure due to ROP, but 34.5% had unfavorable visual outcomes, indicating that other factors such as refractive errors or nystagmus lead to poor visual outcome^13^. McLoone et al. reported 10 eyes with poor visual outcomes, of which only three had poor structural outcomes^14^.

According to previous studies, the prevalence of strabismus in children born preterm ranges from 16 to 22%, which is substantially higher than that in children born at term (1–3%)^15^, and VanderVeen et al. reported that 60% of children with type 1 ROP had strabismus^16^. Previous studies have also shown that preterm babies are more prone to abnormal refractive errors, i.e., myopia, hypermetropia, astigmatism, anisometropia, and strabismus^17^. Moreover, the stage and zone of ROP in patients have a significant effect on myopic progression with severe disease, resulting in faster myopic progression^18^. Therefore, early visual evaluation and management of strabismic or anisometropic amblyopia are needed in preterm babies, especially in children with ROP.

In accordance with previous studies, abnormal refractive errors, anisometropia, and strabismus after ROP regression significantly correlated with poor visual outcomes in our study^19^.

There are some limitations to our study. First, because it had a retrospective design and patients with threshold ROP (who underwent laser coagulation or anti-VEGF treatment) had possibly visited the hospital long enough to assess visual outcome, this study may have included a relatively small proportion of non-treated, less severe patients, therefore leading to some selection bias. Second, for the same reason, we found it difficult to set a control group, leaving this study with limited results. Further prospective studies comparing with a control group (premature babies without a history of ROP) are needed. Third, the age range (2.4 to 9.0 years old) is quite diverse, which would require different efforts on "normal" visual acuities. However, our study may still be meaningful in several aspects in terms of analyzing correlation between retinal anatomical sequelae and long-term visual outcomes in ROP patients.

To summarize, the majority of patients with regressed ROP in our study had favorable visual outcomes, indicating the importance of early management and closely monitored follow-up. However, even after regression with adequate treatment, anatomical and functional sequelae can occur. Our study suggests predicting visual outcomes using macula-to-disc distance in fundus photographs. We also investigated factors related to poor visual outcomes using long-term visual outcome data of patients with ROP after proper management.

## Data Availability

All data relevant to the study are included in the article or uploaded as supplementary information.
